# The need for an equity-focused, trauma-informed PBIS system: addressing the gaps in school discipline

**DOI:** 10.3389/fpsyg.2026.1828592

**Published:** 2026-06-09

**Authors:** Talisa F. Stone-Hausman, Erin R. Yosai, Clarissa Ross, Khushi Patel, Madeline Deasy

**Affiliations:** Department of Educational Psychology, University of Kansas, Lawrence, KS, United States

**Keywords:** discipline, equity-focused, MTSS, PBIS, school, trauma-informed

## Abstract

Schools in the United States have moved from zero-tolerance policies towards MTSS frameworks, like PBIS, but exclusionary discipline and racial/socioeconomic disparities persist, especially for students of color and students with disabilities. While evidence suggests that PBIS is effective in reducing overall discipline rates and positively impacting academics, behavior, and school climate, it fails to address the persistent gap in discipline disparities or the increasing impact of trauma on youth. This paper conducts a narrative review of policies, guidelines, and frameworks related to behavioral interventions and supports in schools, with particular attention to how issues of trauma and equity are addressed. Findings suggest that policies often endorse supportive, multi-tiered frameworks (e.g., PBIS) and trauma-informed practice as separate initiatives and offer minimal practical guidance on implementation. Additionally, while some guidelines and frameworks offer suggestions for implementing PBIS with a trauma-informed or equity lens, these three components are not often discussed together. This paper proposes an integrated trauma-informed, equity-focused PBIS framework that embeds trauma principles and equity strategies across all MTSS tiers and discipline decision points. Actionable recommendations are included for schools and directions for future research and policy are discussed.

## Introduction

School discipline systems in the United States have undergone substantial shifts over the past several decades. Historically, many schools relied on zero-tolerance disciplinary policies designed to deter serious misconduct through predetermined consequences, often including suspension or expulsion. These policies were originally implemented in response to concerns regarding school safety, drugs, and violence; however, research has consistently demonstrated that exclusionary disciplinary approaches are largely ineffective at improving student behavior and may contribute to negative academic and social outcomes (Henry-Hogarth, 2018; Holland, 2019; Henry et al., 2021; Reynolds, 2020). Exclusionary discipline practices have also been linked to increased disengagement from school, higher dropout rates, and involvement with the juvenile justice system (e.g., Hirschfield, 2018).

A growing body of research has also documented persistent racial and socioeconomic disparities in school discipline outcomes. Students of color, particularly Black and Hispanic students, are disproportionately represented in suspension and expulsion rates compared to their white peers (Henry-Hogarth, 2018). These disparities are often driven by subjective disciplinary decisions made by schoolteachers or administration related to behaviors such as “defiance” or “disrespect,” which may be influenced by implicit bias and cultural misinterpretations of student behavior. For example, a teacher can unconsciously interpret repetitive misbehavior like throwing planes by a Black student as defiance and impose a harsh punishment. Another example documented was tardiness by Black students being interpreted as a sign of disrespect, for which suspension from school was reported to be the punishment. This could leave the student feeling unaccepted within the school and cooperating less with teachers. Teachers might then continue to impose harsh punishments, creating an avoidable cycle of disciplinary action (Quereshi and Okonofua, 2017). These persistent disparities helped shift national conversations about school discipline away from purely punitive models and toward prevention-oriented frameworks designed to improve school climate while reducing exclusionary practices (Henry et al., 2021; Liasidou, 2024; Onipede et al., 2024). In response to these concerns, many schools have adopted Multi-Tiered Systems of Support (MTSS) frameworks that emphasize prevention, early intervention, and data-based decision making. Within MTSS, Positive Behavioral Interventions and Supports (PBIS) has emerged as one of the most widely implemented frameworks for promoting positive school climate and addressing behavioral concerns. PBIS focuses on teaching and reinforcing expected behaviors, using data to guide intervention decisions, and implementing tiered supports for students with increasing levels of need. Although these outcomes contributed to PBIS becoming one of the most widely adopted behavioral frameworks in schools, growing awareness of childhood trauma soon raised additional questions about whether behavioral systems alone were sufficient to address students’ underlying emotional and relational needs (Reynolds, 2020; Thomas, 2021; Floress and Jacoby, 2016; Thomas, 2020; Roe, 2022; White, 2021).

At the same time, increasing awareness of the prevalence and impact of childhood trauma has prompted schools to explore trauma-informed approaches to education. Adverse childhood experiences (ACEs), including exposure to violence, abuse, neglect, or chronic stress, can significantly affect cognitive development, emotional regulation, and behavioral functioning (Mahajan, 2018). Trauma-informed school frameworks seek to create environments that emphasize safety, trust, collaboration, and emotional regulation to better support students affected by trauma. As trauma-informed practices gained traction in educational settings, schools increasingly began positioning PBIS and trauma-informed care as complementary approaches to discipline and student support (Substance Abuse and Mental Health Services Administration, 2014; Liasidou, 2024).

Although PBIS and trauma-informed practices are frequently promoted as complementary approaches, existing policy guidance and implementation frameworks often treat them as separate initiatives. In practice, this fragmentation can result in PBIS systems that emphasize behavioral compliance without adequately addressing the underlying impacts of trauma, systemic inequities, and culturally diverse behavioral norms. Critically, Kim and Venet (2023) argue that PBIS itself may function as trauma-inducing, as its emphasis on surveillance, behavioral labeling, and compliance-based reinforcement systems can replicate the same conditions of control and unpredictability that can be harmful to students with trauma histories. Consequently, despite reductions in overall disciplinary incidents, racial and socioeconomic disparities in discipline outcomes frequently persist in schools implementing PBIS (Donelson, 2020; Onipede et al., 2024).

To address these gaps, scholars have increasingly called for PBIS frameworks that explicitly integrate trauma-informed principles and equity-focused practices. Such integration requires attention not only to behavioral interventions, but also to the broader systemic and relational factors that shape how student behavior is interpreted and addressed within school environments (Onipede et al., 2024).

Understanding how current federal, state, and organizational policies and guidance address behavioral interventions in schools, with particular attention to the integration of trauma-informed practices and equity considerations within PBIS frameworks is essential for those involved in the education field. Through a narrative review of policy initiatives and implementation guidance, this paper highlights key strengths, limitations, and gaps in current approaches. Building on this analysis, the manuscript proposes an integrated framework for trauma-informed, equity-focused PBIS implementation and offers actionable recommendations for schools seeking to transform disciplinary systems into environments that promote belonging, safety, and improved outcomes for all students.

### Multi-tiered systems of support (MTSS)

Multi-Tiered Systems of Support (MTSS) provide an organizational framework through which schools deliver academic, behavioral, and social–emotional supports at increasing levels of intensity. MTSS is grounded in prevention science and emphasizes universal supports for all students (Tier 1), targeted interventions for students at risk of behavioral concerns, social–emotional difficulties, or falling behind in academics without additional support (Tier 2), and more intensive and individualized interventions for students with needs not fully addressed through Tier 2 supports (Tier 3). Decision-making within MTSS is guided by ongoing data collection, progress monitoring, and collaborative problem-solving teams that adjust supports based on student response. Because MTSS structures schoolwide systems for identifying needs, delivering interventions, and monitoring outcomes, it serves as a critical infrastructure for integrating behavioral frameworks such as PBIS with other approaches designed to support student wellbeing, including trauma-informed practices.

### Trauma-informed practice

Trauma-informed approaches to education emphasize that exposure to adverse and chronic stressors can shape learning, behavior, and relationships by affecting students’ physiological stress responses, emotional regulation, and sense of safety. Trauma-informed frameworks are commonly grounded in principles such as safety, trustworthiness, collaboration, empowerment, and cultural responsiveness (Substance Abuse and Mental Health Services Administration, 2014; Liasidou, 2024). Rather than functioning as a single program, trauma-informed education is best understood as an organizing approach that influences how adults interpret behavior, how schools structure environments, and how staff respond to student distress. Across tiers of support, trauma-informed practices prioritize predictable routines, relational safety, regulation skills, and strategies designed to avoid re-traumatization (Onipede et al., 2024; Joseph et al., 2020; Liasidou, 2024; Donelson, 2020).

### Positive behavioral interventions and supports (PBIS)

Positive Behavioral Interventions and Supports (PBIS) is a multi-tiered, prevention-oriented framework designed to improve school climate and reduce problem behavior byteaching students expected behaviors through explicit instruction, reinforcing prosocial conduct, and using data to guide decisions and allocate supports. Additional core features include consistent acknowledgement systems and ongoing fidelity monitoring and coaching for all educational professionals. PBIS is typically implemented within a Multi-Tiered System of Support (MTSS) structure, in which Tier 1 universal practices establish schoolwide expectations and routines, Tier 2 provides targeted supports for students requiring additional intervention, and Tier 3 offers individualized, intensive interventions (White, 2021). When implemented with high fidelity, PBIS is associated with reductions in office discipline referrals and improvements in perceived school climate (Reynolds, 2020; Thomas, 2021; Floress and Jacoby, 2016; Thomas, 2020; Roe, 2022; White, 2021).

### The promise and limitation of PBIS

The PBIS approach is rooted in Applied Behavior Analysis (ABA), which emphasizes the use of environmental cues, reinforcement, consequences, and systematic data to shape behavior (Thomas, 2020; Thomas, 2021). PBIS was introduced in schools for the first time in the 1980’s in response to growing concerns regarding behavioral disorders for which behavioral interventions were needed to decrease disruptions in the classroom. It was implemented as a new strategy to deal with behavior problems in a less punitive or stigmatizing way and to create a more positive learning environment (White, 2021). The three-tiered system applied in PBIS emphasizes teaching and reinforcing positive behaviors rather than punishing negative ones. It is designed to promote both academic and social outcomes with the use of evidence-based behavioral practices and positive reinforcement (White, 2021).

Implementing PBIS has been found to decrease rates of exclusionary discipline, improve school climate, increase academic achievement, and decrease student aggression. These findings are supported by a study conducted by Reynolds (2020) who wanted to determine the effectiveness of PBIS. Thomas (2021) also conducted a study to determine the effects of school wide PBIS implementation on academic achievement at an all-male, African American, school. It was found that the implementation of PBIS was positively associated with decreased exclusionary disciplinary action and was linked to an increase in school attendance and a more positive motivation to learn. Another study by Floress and Jacoby (2016) looked at the effects of a specific classroom management strategy that follows the school wide PBIS guidelines. After implementation in a preschool, kindergarten, and second grade classroom, a decrease in disruptive behavior was found as well as an increase in praise from teachers for desired behavior. Thomas (2020) also reviewed the effect of PBIS implementation in a middle school with a history of high discipline rates, making this study more intentional in comparison to the studies previously mentioned by explicitly studying the impact of PBIS in a school with high discipline rates. The approach was also individualized to match the school’s values, expectations and established reward systems. Results of this study found that the implementation of PBIS benefitted the school’s overall positive climate in addition to reducing disciplinary rates. Further, Roe (2022) conducted a study to determine the effects of PBIS intervention after adapting and implementing in a Belizean private school. Things like expected behavior, discipline, culturally specific activities, and decision making were adapted to fit the school’s needs and cultural values. Teachers reported that these adaptations aligned with their school’s cultural values and were perceived as effective in improving school culture. Classroom observations also documented positive student behaviors related to their identified schoolwide expectations, and students appeared responsive to the reward system. These positive effects provide further evidence that PBIS is adaptable, flexible, and scalable to fit different cultural contexts. So, across diverse school settings, PBIS has been associated with improved student behavior, stronger school climate, and measurable academic gains.

To further justify the effects of PBIS, research has suggested that educators generally view PBIS favorably, which is integral for implementation (Floress and Jacoby, 2016). After appropriate training, educators have a good understanding of the core PBIS features while emphasizing multiculturalism, relationships between students and teachers, and promoting positive school-community relationships (Roe, 2022). It is very important that educators are prepared through training and professional development, and continuous, supportive, follow-up meetings with school leadership, building-level PBIS teams, and/or coaches before and during implementation. Positive educator attitudes towards PBIS have been linked to better relationships with students, more proactive actions, less disciplinary action, and higher academic achievement (Thomas, 2021). Further, in a study conducted by Henry et al. (2021), over 70% of educators had heard of zero-tolerance policies, but most reported that they did not agree that these policies were safer or more effective, and about half disagreed that schools should have these policies for misbehavior. These findings suggest that many educators are open to replacing punitive discipline systems with more restorative, supportive frameworks like PBIS. Despite this growing support for PBIS among educators, evidence suggests that implementation alone does not necessarily translate into equitable disciplinary outcomes across student populations.

Currently, PBIS is widely implemented in school environments and has been praised for finding success in reducing overall disciplinary referrals, but this approach often fails to reduce racial and socioeconomic disparities in exclusionary discipline (Donelson, 2020; Onipede et al., 2024). Students from marginalized groups, like Black, Latinx, multilingual learners, and students with disabilities, remain overrepresented in exclusionary disciplinary outcomes (Onipede et al., 2024; Joseph et al., 2020). One reason for this is that PBIS is often implemented as a race-neutral framework, failing to acknowledge how racism and systemic bias influence discipline (Joseph et al., 2020; Donelson, 2020). Without intentional attention towards equity, the standard PBIS framework risks reinforcing disparities instead of eliminating them. These disparities become even more concerning when viewed through a trauma-informed lens, particularly because trauma-related behaviors are often interpreted through culturally and racially biased disciplinary framework (Donelson, 2020; Onipede et al., 2024).

When interpreting student behavior, educators may unintentionally succumb to implicit bias, which unfortunately leads to disproportionate discipline rates for students of color (Joseph et al., 2020; Onipede et al., 2024). Often, trauma-related behaviors can present as hypervigilance, withdrawal, or emotional outbursts. These behaviors may be misinterpreted as defiant or disrespectful, especially when demonstrated by students of color (Liasidou, 2024; Onipede et al., 2024). The assumption that students will respond the same to rewards and consequences is also an underlying feature of the PBIS framework, which ignores the potential impact of trauma on individual learning and behavior (Liasidou, 2024). When students have experienced trauma, relationship-based support and regulation strategies may be helpful for providing emotional safety instead of just relying on behavioral correction. Without these relational and regulatory supports, PBIS systems risk reinforcing punitive responses that may further alienate or retraumatize vulnerable students (Joseph et al., 2020; Onipede et al., 2024).

Certain more punitive practices used within the PBIS framework, like restraint or seclusion, can retraumatize students who have already lived through adverse experiences or who currently experience chronic stress (Liasidou, 2024). PBIS often emphasizes rule-following over emotional support, which can foster a compliance culture that prioritizes order over feelings of safety or belonging (Donelson, 2020; Liasidou, 2024). In schools that are under-resourced, PBIS may contribute to an environment with hyper-surveillance, which can result in students (especially students of color) feeling constantly monitored, data-tracked, and punished for minor infractions, which is exactly what PBIS is trying to avoid (Sheikh et al., 2024). These high surveillance environments can feel punitive and alienating, especially for students who are already feeling vulnerable due to systemic discrimination (Joseph et al., 2020; Sheikh et al., 2024). To disrupt these harmful patterns, PBIS must be reimagined as a system that centers equity, trauma-awareness, and cultural responsiveness.

### Where MTSS, PBIS, and trauma-informed practice align

PBIS and trauma-informed approaches share important points of convergence. Both emphasize prevention, proactive teaching, and multi-tiered supports that match intervention intensity to student need. Both aim to strengthen school climate and reduce reliance on punitive discipline. Additionally, trauma-informed practice can be integrated into PBIS structures by strengthening Tier 1 relational practices and regulation supports, refining Tier 2 targeted interventions for students experiencing distress, and ensuring Tier 3 individualized plans incorporate mental health and wraparound supports when needed In this sense, PBIS offers an implementation architecture (systems, teams, routines, data), while trauma-informed education offers interpretive and relational guidance for responding to behaviors rooted in stress and adversity. However, conceptual alignment does not necessarily translate into cohesive implementation, particularly when discipline decisions are filtered through implicit bias, compliance-oriented systems, and culturally narrow behavioral expectation.

### Divergence at the point of discipline

Despite conceptual compatibility, PBIS and trauma-informed approaches are frequently implemented as separate initiatives, and their divergence often occurs at the most consequential point in school discipline: adult interpretation and decision making (Eber et al., 2020; Onipede et al., 2024). PBIS implementation can drift toward a compliance-oriented model if behavioral expectations reflect dominant cultural norms for communication and emotion, or if reinforcement/response systems emphasize rule-following over relationship and regulation. Trauma-informed practice may similarly remain superficial when schools adopt the language of trauma-informed care without building operational systems for training, coaching, data review, and accountability (Center on Positive Behavioral Interventions and Supports, 2023; Eber et al., 2020). These implementation pitfalls are not neutral: discipline decisions are disproportionately applied to students of color and other marginalized groups, particularly in subjective categories such as defiance or disrespect. When trauma-related behaviors are misinterpreted as willful misconduct, especially in contexts shaped by implicit bias, students may experience punitive responses rather than supportive intervention. As a result, schools may observe overall decreases in referrals while disproportionality in exclusionary discipline persists (Donelson, 2020; McIntosh et al., 2021; Onipede et al., 2024).

Taken together, the literature suggests that the challenge is not whether PBIS and trauma-informed practice can coexist, but whether schools are equipped to integrate them in ways that explicitly address equity, interpretation, and disciplinary decision making. Because these frameworks intersect most clearly at the level of implementation, understanding how current policies and guidance conceptualize trauma, behavioral support, and equity becomes essential. The following section therefore examines how federal and organizational policies have approached PBIS, trauma-informed practice, and discipline reform within contemporary school systems.

## Federal guidance on school discipline

Because PBIS and trauma-informed practices are frequently promoted within educational policy and guidance documents, understanding how these frameworks are represented across federal and organizational initiatives is essential. The following section reviews key policies, guidance, and frameworks that shape how schools implement behavioral supports and trauma-informed approaches.

### Department of Education

The U.S. Department of Education (DOE) has recommended the use of PBIS as an evidence-based, multi-tiered framework designed to improved school climate, student behavior, and overall learning conditions. The DOE has awarded over $226 million since 2014 to the School Climate Transformation Grant Program, whose goal is to help states and districts build and strengthen their PBIS systems in their schools. Additionally, a national PBIS Technical Assistance Center is funded to help schools, districts, and states effectively implement tiered social, emotional, and behavioral supports (Federal Commission of School Safety, 2018). Federal guidance from the U.S. Department of Education (DOE) has also played a critical role in shaping school discipline, especially in relation to conversations about equity. The DOE has focused on school climate and student discipline resources rooted in social, emotional, behavioral, and mental health needs (School Climate and Student Discipline Resources, 2023).

Federal guidance has increasingly emphasized prevention-oriented approaches to school discipline that prioritize positive school climate, student engagement, and the integration of social, emotional, and behavioral supports. These efforts align with broader federal initiatives focused on addressing student mental health needs and promoting equitable disciplinary practices across schools (School Climate and Student Discipline Resources, 2023).

The PBIS Implementation Blueprint further highlights that effective implementation requires coordinated infrastructure across school, district, and state systems to sustain multi-tiered behavioral supports (Center on Positive Behavioral Interventions and Supports, 2023). This infrastructure includes leadership teams, professional development systems, coaching supports, and ongoing data-based decision-making processes that guide implementation and monitor outcomes. Within this framework, schools establish clear behavioral expectations, explicitly teach and reinforce prosocial behaviors, and use discipline data to inform continuous improvement efforts. Importantly, the Blueprint emphasizes that PBIS is not a standalone program, but a systems-level prevention framework designed to organize evidence-based behavioral practices and ensure consistent, equitable implementation across educational contexts. Although these frameworks provide important structural guidance, they offer comparatively limited direction regarding how schools should operationalize trauma-informed and equity-focused discipline practices within day-to-day implementation.

### Supportive School Discipline Initiative

When addressing behavioral concerns in schools, discipline data and policies are also considered, especially since there are persistent disparities in discipline. In 2011, the Federal government announced its efforts to battle the school-to-prison pipeline and persistent racial inequities with the Supportive School Discipline Initiative. The Supportive School Discipline Initiative (SSDI) represents the joint efforts of the U.S. Department of Education (DOE) and the Department of Justice to address inappropriate school discipline, like the reliance on zero-tolerance policies. SSDI specifically began as a focus of racial equity by acknowledging disproportionate discipline as an issue of civil rights (Supportive School Discipline Initiative, 2014). However, these recommendations were nonbinding and failed to address the root causes of racial inequity within discipline policies. Meaningful change cannot occur without understanding the effects of implicit bias among faculty and educators, the inadequate resources in underfunded schools, and broader structural inequalities. Effective interventions would require need-based education reform, explicitly addressing racial inequity, and new economic policies that are targeted to help lower income communities (Spiller and Porter, 2014). Within this framework, trauma-informed care is also addressed implicitly rather than explicitly, offering limited guidance on how these practices should be implemented. Since its establishment, the initiative prompted effective alternatives to exclusionary discipline with an emphasis on reducing disproportionality among students with color and with disabilities (Supportive School Discipline Initiative, 2014). However, in 2018, the Trump administration issued a withdrawal of such “harsh” guidance and as of 2025, the White House pushed a return to greater local control over discipline policy (White House, 2025). These shifts highlight the instability of federal discipline guidance and reinforce the importance of developing implementation frameworks that remain responsive to equity and trauma concerns regardless of changing political priorities.

### Every Student Succeeds Act

The Every Student Succeeds Act (ESSA) is a federal law that was first enacted in the 2017–2018 school year in efforts to increase comprehensive psychological services access in schools by requiring K-12 educational agencies to invest in evidence-based practices. ESSA encourages the state to adopt evidence-based interventions and accountability systems using a Multi-Tiered System of Support (MTSS) framework, like Positive Behavioral Interventions and Support (PBIS) (ESSA Overview for School Psychologists, 2018). This framework supports an inclusive and developmentally oriented program that seeks to improve school climate, safety, and access to high quality comprehensive learning support. More specifically, ESSA supports state and district efforts in screening, early intervention, improvement of quality and effectiveness of family and community partnerships, and more relevant training for school staff (ESSA Overview for School Psychologists, 2018). ESSA also signals support for trauma-informed practices, particularly through its emphasis on student mental health, social–emotional learning, and school climate.

### National Child Traumatic Stress Network

Growing recognition of trauma’s short- and long-term effects (e.g., behavior, learning, development), and its prevalence, has led to local, state, and federal legislation and policies to address the importance of making systems trauma-informed to help address trauma-related behaviors and symptoms in schools. Between the years of 1973 and 2015, roughly 49 bills within federal legislation mentioned “trauma-informed practice,” including the ESSA and SSDI previously mentioned. Many of these bills were specifically referring to youth in K-12 schools and organizations. Due to this growing awareness, the National Child Traumatic Stress Network (NCTSN), funded by SAMHSA, was created to provide extensive training and technical assistance on trauma-informed practices in educational settings. This federally funded child mental health initiative emphasizes the impacts of trauma, secondary traumatic stress among educators, and the importance of safe, supportive school environments on the development of children in K-12. The framework specifically addresses 10 core areas of focus: Identification and Assessment of Traumatic Stress, Prevention and Intervention Related to Traumatic Stress, Trauma Education and Awareness, Partnerships with Students and Families, Creation of a Trauma-Informed Learning Environment, Cultural Responsiveness, Emergency Management/Crisis Response, Staff Self-Care and Secondary Traumatic Stress, School Discipline Policies and Practices, and Cross System Collaboration and Community Partnerships (The National Child Traumatic Stress Network, 2018). This framework defines Trauma-Informed School (TIS) as one that integrates trauma-informed approaches into a holistic view of school systems to enhance student outcomes, and it integrates actionable resources to motivate Trauma-Informed Schools. While the framework provides a comprehensive vision for trauma-informed schools, questions remain regarding how these principles can be systematically embedded into existing behavioral infrastructures such as PBIS.

### IDEA law

The Individuals with Disabilities Education Act (IDEA) is a federal law to ensure that children with disabilities receive free appropriate public education (FAPE) by mandating special education, early intervention, and support for families. This Act therefore promotes an education that has a less restrictive environment but also tailored to the needs of children with disabilities. As a federal law, the Department of Education provides resources to assist schools in the implementation. As a part of IDEA’s disaster response efforts, the Office of Special Education Programs (OSEP) provides guidance under IDEA that focuses on the Positive Behavioral Interventions and Support Center (PBIS) for crisis recovery (IDEA Topic Areas, 2025). This refers to the PBIS website to support the MTSS framework in Crisis Recovery (n.d.). Furthermore, it derives from the National Center on Safe and Supportive Learning Environments for trauma recovery, which focuses on trauma-sensitive schools. This website provides a training package, resilience toolkit, and additional resources to integrate trauma-informed care into the recovery environment (Supporting Trauma Recovery, n.d.).

### Trauma-informed PBIS within MTSS

PBIS, as is, does not always account for how past experiences with trauma influence behavior, especially in students of color who are at higher risk of exposure to adverse experiences. Specifically, marginalized groups are at greater risk of experiencing discrimination, poverty, and systemic violence, conditions that may be perceived as traumatic (Joseph et al., 2020; Onipede et al., 2024). In schools, behaviors perceived as defiant or noncompliant may just be trauma induced hypervigilance, emotional outburst, or withdrawal. This results in punishment instead of support from adults (Liasidou, 2024; Onipede et al., 2024). Although there is increased awareness surrounding trauma-informed care, educators often receive limited training in trauma-informed or culturally responsive behavior interpretation, resulting in continued bias towards behavior interpretation and intervention delivery (Joseph et al., 2020). This leads to a cycle where students with trauma aren’t supported, which worsens mental health and academic outcomes. These patterns suggest that trauma-informed implementation cannot simply function as an add-on to PBIS but instead requires reexamining how behavioral systems themselves interpret and respond to student behavior (Liasidou, 2024).

Integrating trauma-informed practices into PBIS requires more than adding new interventions; it involves re-examining how behavioral expectations, data systems, and intervention strategies operate within the MTSS structure. MTSS provides a flexible framework in which trauma-informed practices can be embedded across tiers of support. At the universal level (Tier 1), schools can emphasize predictable routines, relational safety, and culturally responsive expectations. Targeted interventions (Tier 2) can incorporate regulation strategies and skill-building supports for students experiencing stress or adversity, while intensive interventions (Tier 3) may involve individualized plans that include mental health collaboration and wraparound services. In this way, trauma-informed practices function as an interpretive and relational lens that strengthens the behavioral infrastructure already established through PBIS. Accordingly, several scholars and implementation frameworks have begun outlining how trauma-informed principles might be operationalized within PBIS system.

To better address the increasing prevalence of trauma in school-aged children, the Center on PBIS provided some recommendations on how to integrate trauma-informed features into a PBIS framework (Eber et al., 2020). They provided recommendations on how to incorporate trauma-informed enhancements into six core MTSS features. The first recommendation is to ensure someone with trauma expertise is involved in MTSS leadership teams to help guide implementation of trauma-informed practice. Secondly, it is recommended that extended data (e.g., ACES data) is used to identify students who are at risk or who are currently experiencing trauma responses. The third recommendation is to ensure schools are using universal screening measures to identify students who may need support. The fourth recommendation encourages schools to select evidence-based trauma-informed practices through a formal process, including expanding evidence-based practices currently being implemented in the school. The fifth recommendation includes deciding how fidelity and effectiveness will be assessed prior to implementation. The last recommendation encourages the use of ongoing professional development and coaching. In addition to these recommendations, they include tables with examples of how to adjust Tier 1 expectations and strategies for staff members. While this resource provides explicit recommendations and examples of how to integrate trauma-informed practices into PBIS frameworks, it is non-binding. Additionally, given that standard PBIS implementation varies across districts and states in both policy and practice, trauma-informed PBIS likely faces even greater inconsistency, as it is an emerging integration with no established policy infrastructure (Molloy et al., 2013; Center on Positive Behavioral Interventions and Supports, 2026). Beyond implementation inconsistency, scholars have also raised concerns regarding the cultural assumptions embedded within many traditional PBIS models.

PBIS frameworks also often use standardized behavioral expectations and reinforcement systems that reflect white, middle-class norms for communication, emotion, and compliance (Joseph et al., 2020). This race-neutral implementation strategy fails to question the diversity of norms across cultures and populations, which results in discipline structures that privilege whiteness (Donelson, 2020). Drawing from Critical Race Theory (CRT), this reflects the concept of “whiteness as property”, which was originally theorized by Harris (1993) as the way whiteness functions as a form of privilege codified within institutional systems. This includes school norms that are frequently created around dominant cultural values, resulting in deviations being pathologized (Joseph et al., 2020). So, when PBIS is not race-conscious and equity-focused, there is risk of reinforcing this whiteness hierarchy and further deepening disparities (Donelson, 2020; Onipede et al., 2024).

### Embedding equity into behavioral systems

A randomized control trial conducted by McIntosh et al. (2021) found evidence to support that an equity-focused PBIS approach helps reduce racial inequities in school discipline. They developed an adapted PBIS training approach for school staff based on, “(a) elements of PBIS that are most strongly related to racial equity in school discipline, (b) a theory of the operation of implicit bias in school discipline decision making, and (c) research on increasing fidelity of school-based interventions.” This approach was labeled ReACT (Racial equity through Assessing data for vulnerable decision points, culturally responsive behavior strategies, and teaching implicit bias and how to neutralize it) and was created to as a professional development tool for school staff. ReACT included commitment statements from administrators, personal examples of implicit bias, choice in implementation strategies, testimonials from school staff that piloted the implementation strategies, and ongoing coaching from the school PBIS team.

The first component of ReACT is “Assessing data for vulnerable decision points”. This involves identifying current racial/ethnic inequities in their school discipline data and analyzing when, where and why these inequities are more likely to occur. For example, they try to identify what situations bias is more likely to influence discipline decisions (e.g., black students getting more defiance referrals). The second component is “culturally responsive behavior strategies”. This involves selecting which intervention strategies from a pre-established menu are most likely to address the identified inequities while still aligning with staff skills and values. These strategies include things to help improve student-teacher relationships, teaching desired behaviors, and how to respond to unwanted behaviors. The third component is “teaching about implicit bias and how to neutralize it”. This involved professional development about implicit bias and how it can influence discipline decisions. Staff also learn neutralizing routines to help replace snap-judgement decisions regarding in-the-moment discipline decisions.

After a year of implementing ReACT, results found evidence of statistically significant improvements in the racial disparities in school discipline data for the intervention schools, while the control schools showed no improvement. While this is preliminary data from only one school district, it helps provide some meaningful contributions to research on how to effectively address racial disparities within current widespread intervention frameworks (i.e., PBIS) (McIntosh et al., 2021). While the results of this study are encouraging, the information is not widespread as there is only one published study available, which means that these strategies are not currently being encouraged or implemented in schools outside of research. However, there is currently an ongoing 5-year (2023–2028) randomized control trial underway evaluating the effects of ReACT on racial disparities in discipline, academic achievement, and school climate across multiple schools (Institute of Education Sciences, 2023).

## Recommendations

### Practical recommendations for schools

Research has found that PBIS is effective in reducing overall discipline referrals and increasing positive behavior and school climate. Preliminary research has also shown promising evidence for using professional development focused on racial equity to help reduce racial disparities in discipline. The next step is to effectively address the impact of trauma on students’ behavior, learning, and development. A PBIS model that is redesigned to go beyond surface-level reform by embedding trauma-informed principles and racial equity into all tiers of intervention could address the lingering disparities within school systems (Liasidou, 2024; Joseph et al., 2020). According to Onipede et al. (2024), core elements of a new PBIS model should include behavioral expectations and reinforcements that are culturally relevant, Tier 1 practices for emotional regulation and social–emotional learning (SEL), restorative practices in response to conflict, and disaggregated discipline data monitoring to flag disparities continuously. Ongoing training should also be required and offered for teachers and staff regarding trauma and implicit bias. This can be completed by educating staff on the effects of trauma on a child’s wellbeing. One strategy is to encourage staff to ask, “What are this child’s lived experiences?” and “Are their behaviors related to those experiences?” rather than “What’s wrong with this child?” and “Why won’t this child just do what I’m telling them” (Pastrana Rivera et al., 2025)? This will help ensure interventions are supportive and not punitive (Joseph et al., 2020; Donelson, 2020). With good implementation fidelity, a new model with these core elements could reduce disproportionality, improve student-teacher relationships, and promote long-term emotional wellbeing (Onipede et al., 2024). Translating these principles into practice, however, requires schools to operationalize trauma-informed and equity-focused supports across each tier of the MTSS framework.

While several frameworks have begun to outline how trauma-informed principles can be incorporated into PBIS systems, implementation guidance remains inconsistent across districts and states. The following recommendations outline practical strategies schools can use to integrate trauma-informed and equity-focused practices across MTSS tiers. A trauma-informed framework should be grounded in core principles of safety, trustworthiness, collaboration, empowerment, and cultural responsiveness (Substance Abuse and Mental Health Services Administration, 2014; Liasidou, 2024). These principles help shift the school environment’s focus on prioritizing connection, emotional regulation, and inclusive support instead of focusing solely on behavioral compliance (Joseph et al., 2020). Trauma-informed systems avoid retraumatizing students by instead creating space for emotional recovery and growth, especially for students that have experienced ACEs (Onipede et al., 2024). These core principles should be reflected in the climate, policies, and responses across all PBIS tiers within an MTSS framework.

Tier 1 represents schoolwide or universal strategies that support all students. Regarding behavior within a PBIS system, there are multiple ways PBIS can be adjusted to incorporate a trauma lens. For example, student-teacher relationships can be built daily through morning check-ins or greeting students as they come into the classroom. Emotional regulation and SEL curriculum could be embedded in the general school curriculum. Routines that are predictable and have flexible expectations can also be beneficial in reducing anxiety among students (Liasidou, 2024; Jacobson, 2021). Language used by all staff should avoid deficiency driven terms such as: “unwanted behavior” and “problem behavior” (Kim and Venet, 2023). Instead creating language and expectations using affirming phrases such as: “follow directions given the first time” or “creating a positive atmosphere.” Considering students’ needs as more important than adults will help staff avoid “hyper-punishment,” that often leads to an increased rate of discipline referrals (Kim and Venet, 2023). Another effective strategy is to use “person centered planning” for students requiring interventions (Kim and Venet, 2023). With proper training, school staff should also be able to recognize behavioral signs of trauma and respond with empathy instead of punishment (Donelson, 2020). While these universal supports may strengthen relational safety for all students, some students experiencing chronic stress or trauma exposure may require more targeted intervention.

Tier 2 supports are more targeted, and within a trauma-informed PBIS system, these supports should include supports for students showing signs of trauma-related distress or behavioral dysregulation. Interventions may include small group social–emotional skill building, a mentoring system, a check-in/check-out system with a trusted adult, short-term counseling, or trauma-specific support groups (Onipede et al., 2024). Supports should become more individualized and must be responsive to students’ cultural and racial identity since trauma is often experienced differently across groups (Joseph et al., 2020). For students with more significant trauma histories or chronic behavioral needs, even more individualized and intensive supports may be necessary.

Tier 3 supports are the most individualized and intensive interventions. Within a trauma-informed PBIS system, these interventions would be for students with significant trauma histories or chronic behavioral challenges. Some of these students may benefit from individualized behavior plans (IEP) and/or a Behavior Intervention Plan (BIP) that are developed with student and caregiver input. In-school or community-based mental health professionals may step in to help students experiencing distress. Wraparound services and collaboration with other professionals and caregivers may also be necessary (Joseph et al., 2020; Liasidou, 2024). Students receiving these Tier 3 services often require long-term support to build relational and environmental safety that account for emotional triggers and regulation strategies.

To address the risk of replicating the same disparities that persist with traditional PBIS frameworks, a trauma-informed PBIS framework must be implemented with explicit attention towards equity (Donelson, 2020; Joseph et al., 2020). Implicit bias is a big roadblock for successfully addressing these disparities because it affects how behaviors are interpreted and which students are referred for intervention and discipline. To address implicit bias among school faculty, ongoing professional development should be given in cultural competence. Discipline data should also be continuously monitored and reviewed to map out changes in disparities and reflect on the discipline process, policies, and procedures (Onipede et al., 2024). Behavioral expectations, the other main component of a PBIS framework, must be culturally responsive and co-created with the communities they serve, including minority communities, so that they aren’t rooted in white, middle-class norms (Joseph et al., 2020). When PBIS systems are grounded in trauma-informed principles and equity, they have the power to reduce discipline disparities and foster environments that promote healing, trust, and growth among all students.

## Future directions toward transformative discipline reform

Systemic level change will require more research, intervention development, and scaling. Longitudinal research on outcomes of equity-centered, trauma-informed PBIS frameworks will be beneficial for progress monitoring and analysis of long-term effects. Implementation models should also be created, embedding critical race theory and trauma-informed practices into a PBIS framework so it can be widespread and easily implemented in schools. Future policy should also address the structural inequities that persist in school funding, staffing, and access to mental health resources. As new PBIS frameworks are created and implemented, the voices of students and families should help shape the new interventions. Although these are not immediately actionable steps, they represent a broader shift towards a solution.

In terms of policy, the gap between more progressive discipline policies and real-world implementation fidelity needs to be addressed. Policy often acknowledges equity and trauma without ensuring accountability for addressing these concerns. More specifically, schools need to not only encourage the use of trauma-informed language and offer professional development, but they also need to support the use of operationally defined trauma-informed care. Schools and professionals also need to advocate for policies that fund training, allow for flexibility in PBIS implementation, and mandate equity through auditing. Overall, there needs to be a push for system-level change, not just surface level reform.

Lastly, within schools, educators, administrators, and policymakers need to critically analyze their own systems and beliefs. They need to look at their school system(s) and analyze their discipline data. Who is getting disciplined most and why? They need to question how their behavioral expectations/norms were defined. Lastly, they need to determine whether they are currently fostering different cultural identities or seeing differences as risks. The staff within schools need to see themselves as capable of making a change. Addressing discipline disparities is about more than managing behavior. It is about transforming school systems into places that make students feel valued, understood, and supported. PBIS has the potential to be successful for all students if we are willing to reimagine its framework through the lenses of trauma and equity.

### Future directions for research and policy

Overall, federal, state, and organizational policies and guidelines encourage supportive discipline, using behavioral frameworks like PBIS, and incorporating trauma-informed practices in schools. These patterns within behavioral policies and guidelines reflect momentum towards addressing the growing behavioral concerns in schools, but they often lack actionable recommendations on what specific implementation strategies should be used across tiers and how to make schools more trauma informed. This results in variability in implementation, fidelity, and effectiveness in addressing all behavioral concerns, the impacts of trauma on students, and the persistent disparities in discipline. Endorsing PBIS and trauma-informed practice while specifying equity-focused, trauma-informed implementation within PBIS allows schools to unconsciously reproduce disparities while appearing to be compliant with best practices. It is also important to note that according to a brief by National Association for the Education of Young Children (2025), the current federal administration has advised the Department of Education to halt its consideration and efforts to address the persistent racial disparities in school discipline. This means that the schools and districts have an even greater responsibility in making sure they acknowledge and address their own racial disparities. These policy and implementation gaps become particularly important when considering the growing body of research examining both the strengths and limitations of PBIS implementation in contemporary schools.

Research on the impact of PBIS implementation in schools suggests that PBIS reduces overall discipline referrals, and increases student engagement, positive behavior, and teacher-student relationships. Since traditional PBIS often uses a race-neutral framework and focuses on behavioral compliance, disproportionality in discipline for marginalized communities persist. The lack of consistency in implementing trauma-informed practices into current MTSS frameworks also fails to address the increasing prevalence of trauma among youth. In higher surveillance environments, students’ trauma-related behaviors or symptoms may be exacerbated, or students may experience re-traumatization. Taken together, these findings suggest the need for a framework that moves beyond compliance-oriented discipline models toward systems that explicitly integrate trauma responsiveness and equity-focused implementation.

The proposed recommendation for an equity-focused, trauma-informed PBIS framework combines components of equity-focused PBIS and recommendations for trauma-informed PBIS to explicitly address the persistent disparities in discipline and the impact of trauma on youth. This framework uses the MTSS architecture (tiers, data, teams) to embed trauma principles (e.g., safety, trust, collaboration, empowerment, cultural responsiveness) and equity strategies (e.g., implicit-bias training, neutralizing routines, culturally responsive expectations, disaggregated data review) directly into decision points. Immediate actionable recommendations for schools include professional development for implicit-bias and trauma, revising the language and expectations in the school, integrating more SEL and regulation into Tier 1, embedding more specific trauma supports into tier 2–3, and routinely auditing discipline data. Although these recommendations provide a foundation for immediate systems change, continued research and policy development will be necessary to strengthen implementation fidelity and long-term sustainability.

In the future, increasing research that focuses on how to effectively implement equity-focused and trauma-informed PBIS models into preexisting PBIS or MTSS frameworks will be key to successful systems implementation. The impact on student outcomes and disproportionality should be analyzed to determine what strategies, interventions, supports, and professional development are needed to successfully address persisting disproportionalities and the impact of trauma on youth. Additionally, while some policies need to allow room for flexibility, school, district, state, and/or organizational level policies and guidelines should provide more resources and explicit recommendations for how to implement recommended practices effectively and with fidelity. Instead of a policy only saying, “use trauma-informed practices,” there should be more explicit guidelines/requirements, easily accessible resources, and detailed recommendations.

There are a few limitations that should be considered. First, this is a narrative, United States focused policy review that may not capture all the implementation efforts at state, district, or school levels. Secondly, research on how trauma-informed practice and/or equity principles should be embedded into PBIS frameworks, as well as the effectiveness of these combined framework components, is gaining more attention and is adding to the currently limited research. So, as new research comes out and as changes occur within the federal administration, we may continue to see changes in policies, guidelines, and implantation across federal, state, organizational, and local levels. For now, addressing discipline disparities and the impacts of trauma on youth requires reimagining PBIS as a trauma-responsive, equity-centered system so schools become places where all students feel safe, valued, and supported. Ultimately, the limitations of current discipline reform efforts underscore the need to rethink not only how schools manage behavior, but how educational systems conceptualize safety, belonging, and student support.

School discipline is not simply a matter of behavior management; it reflects how educational systems define safety, belonging, power, and student worth. Although PBIS has contributed to important reductions in exclusionary discipline and improvements in school climate, the persistence of racial, socioeconomic, and disability-related disparities suggests that implementation fidelity alone is insufficient when broader systemic inequities remain unaddressed (Donelson, 2020; Joseph et al., 2020; McIntosh et al., 2021; Onipede et al., 2024). Current educational policy increasingly encourages schools to adopt preventive, multi-tiered, and trauma-informed approaches to discipline; however, policy guidance frequently stops short of providing operational frameworks capable of meaningfully addressing trauma, implicit bias, and inequitable disciplinary decision-making in practice (ESSA Overview for School Psychologists, 2018; Supportive School Discipline Initiative, 2014; The National Child Traumatic Stress Network, 2018). As schools continue to confront rising behavioral and mental health concerns, increasingly diverse student populations, and ongoing disparities in disciplinary outcomes, frameworks that emphasize compliance without contextual understanding are no longer sufficient (Joseph et al., 2020; Kim and Venet, 2023; Liasidou, 2024). Reimagining PBIS through trauma-informed and equity-focused lenses offers an opportunity to move beyond surface-level reform toward systems that prioritize relational safety, cultural responsiveness, emotional regulation, restorative support, and data-informed accountability (Eber et al., 2020; Onipede et al., 2024). Achieving this transformation will require sustained investment in professional development, culturally responsive implementation practices, disaggregated discipline data review, and systems-level accountability structures that center equity across all tiers of support (McIntosh et al., 2021; Onipede et al., 2024). Ultimately, transforming school discipline requires more than changing interventions; it requires changing how schools interpret behavior, respond to adversity, and define student success (Joseph et al., 2020; Kim and Venet, 2023). When implemented with intentionality and accountability, an integrated trauma-informed, equity-focused PBIS framework has the potential to shift schools from systems of surveillance and exclusion toward environments where all students feel safe, valued, understood, and supported (Donelson, 2020; Liasidou, 2024; Onipede et al., 2024).
